# Design of Dielectric Resonator Antenna Using Dielectric Paste

**DOI:** 10.3390/s21124058

**Published:** 2021-06-12

**Authors:** Hauke Ingolf Kremer, Kwok Wa Leung, Wai Cheung Wong, Kenneth Kam-Wing Lo, Mike W. K. Lee

**Affiliations:** 1 Department of Electrical Engineering, City University of Hong Kong, Hong Kong 999077, China; hikremer2-c@my.cityu.edu.hk (H.I.K.); eewcwong@cityu.edu.hk (W.C.W.); wkmikelee@gmail.com (M.W.K.L.); 2 Department of Chemistry, City University of Hong Kong, Hong Kong 999077, China; bhkenlo@cityu.edu.hk

**Keywords:** dielectric resonator antenna, dielectric paste, material measurement, wideband antenna, circularly polarized antenna, fractal antenna

## Abstract

In this publication, the use of a dielectric paste for dielectric resonator antenna (DRA) design is investigated. The dielectric paste can serve as an alternative approach of manufacturing a dielectric resonator antenna by subsequently filling a mold with the dielectric paste. The dielectric paste is obtained by mixing nanoparticle sized barium strontium titanate (BST) powder with a silicone rubber. The dielectric constant of the paste can be adjusted by varying the BST powder content with respect to the silicone rubber content. The tuning range of the dielectric constant of the paste was found to be from 3.67 to 18.45 with the loss tangent of the mixture being smaller than 0.044. To demonstrate the idea of the dielectric paste approach, a circularly polarized DRA with wide bandwidth, which is based on a fractal geometry, is designed. The antenna is realized by filling a 3D-printed mold with the dielectric paste material, and the prototype was found to have an axial ratio bandwidth of 16.7% with an impedance bandwidth of 21.6% with stable broadside radiation.

## 1. Introduction

Dielectric resonator antennas (DRA) have been researched extensively for the last three decades since their first introduction in 1983 [1]. Some of the main features, which distinguish DRAs from other types of antennas, are their ease of excitation, comparably wide bandwidths, and compact size [2,3]. Owing to manufacturing and material tolerance related issues, DRAs have not found widespread application outside of the academic world. For example, many DRAs employ very special geometric shapes, such as flipped staired DRAs, H-shaped DRAs, T-shaped DRAs [4,5,6], or employ different dielectric constants, such as multilayer ring DRAs or perforated DRAs [7,8,9]. Other structures may employ even more exotic structures, such as hook shaped DRAs [10], Y-shaped DRAs [11], pixelated DRAs [12], multiple circular sector DRAs [13], or have notches [14] and hollow regions in their DRA structures [15]. These structures, even though they may possess remarkable features, such as wide bandwidths, high gains, or multi-band operation, may not be easily manufactured using common methods and materials. Recently, compound dielectric materials have attracted a lot of interest due to the possibility of their unconventional use. For example, in [16], a printable dielectric ink was developed, which was loaded with ferroelectric nano-particles. Using this ink, a fully printable phase shifter could be constructed. This new approach can help to avoid the use of expensive substrates, which are commonly used for such applications. Other examples of compound materials include conductive materials, which can be used for flexible or conformable antenna designs, such as patch antennas [17], flexible antenna substrates with adjustable dielectric constant [18], ceramic epoxy resin composite materials used for antenna miniaturization [19], or flexible magnetic polymer composite substrates with equal permittivities and permeabilities for antenna miniaturization [20]. Despite the obvious advantage of sparing manufacturing cost, another advantage of such compound materials is that they can typically be tuned according to the need. This is especially useful for research and development activities.

In this publication, a new method for DRA manufacturing is investigated. The method utilizes a dielectric paste that is realized by mixing silicone rubber and barium strontium titanate (BST) nanoparticles. The dielectric constant of the dielectric paste can be controlled flexibly by the content of the BST particles inside the paste. The paste is then used to design a circularly polarized DRA with wide bandwidth. A mold is constructed using the 3D-printing technique and then subsequently filled with the dielectric paste material. This way, geometric shapes that cannot be manufactured using conventional approaches can now be realized.

For example, certain antenna types, such as fractal antennas, may require exotic shapes. As fractal antennas are known to be able to increase the bandwidth of antennas significantly, they have been investigated by researchers in the past [21]. For instance, fractal patch antennas have attracted a lot of attention [21,22,23]. Fractal DRAs have been investigated in the past as well [24,25,26]. The realization of fractal DRAs appears more challenging in practice when considering structures such as the Minkowski fractal DRA [24]. However, these types of fractals can be realized using patch antennas quite easily, as the PCB copper etching process can be utilized. Using the dielectric paste approach, new classes of DRAs could be realized. In particular, structures with complicated features or features that are located on the inside of the DRA could be realized, which would otherwise be very challenging to do using common manufacturing approaches. In this publication, a menger sponge fractal structure will be analyzed in order to demonstrate the usefulness of the dielectric paste manufacturing method. The fractal structure is used to design a circularly polarized antenna. A 3D-printed mold is utilized in order to contain the dielectric paste material, which fulfills the function of the DRA. The proposed antenna can be deployed for 2.4 GHz WiFi, ISM, and WiMAX applications. All the simulated results in this publication are generated using HFSS full wave simulation tool.

## 2. Dielectric Paste and Characterization

**Figure 1:**
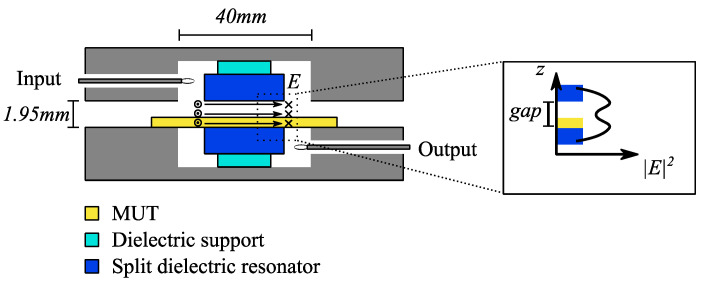
Sketch of the split-post dielectric resonator measurement setup. The MUT is placed in a gap, which separates the dielectric resonator. The electric field in this gap is strong, therefore, the device is sensitive to a change of dielectric constant introduced by the MUT. The change of the field strength along the gap inside the resonator is displayed as well. The E-field is strongest at the edges of the gap close to the resonator and weakest in the center of the gap.

In this section, the process of fabricating and characterizing the dielectric paste will be described. The dielectric paste consists of nano particle-sized barium strontium titanate (BST) powder with a measured dielectric constant and loss tangent of 20 and 0.023, respectively. The BST powder is mixed with a silicone rubber with a dielectric constant of 2.82 and a loss tangent of 0.0215. By changing the BST content relative to the silicone content, the dielectric constant of the paste can be varied according to the need. A split-post dielectric resonator is used to characterize the dielectric paste material. In this measurement, the resonance frequency and insertion loss of a single dielectric resonator (DR) element is measured. The setup is illustrated in Figure 1. The dielectric resonator is embedded into a cavity, and a circular loop is used to excite DR resonance with a second loop serving as the signal output. The DR is split in half, and a gap of approximately 1.95 mm exists between the lower and upper half of the resonator with a minimum sample size of at least 40 mm × 40 mm. This gap can be used to insert a material under test (MUT) and characterize its dielectric properties. The electric field is strong in the gap of the DR, hence, the setup is sensitive to a change of dielectric constant and loss tangent. By measuring the insertion loss and the resonance frequency of the DR before and after the MUT is inserted, the dielectric constant and loss tangent can be extracted.

Under normal operation, the dielectric resonator would be calibrated by measuring the resonance frequency and insertion loss of the measurement device without any sample inserted into the gap at first. As will be shown later, for the dielectric paste measurement, a sample holder was used. In particular, a thin layer of a low loss plastic sheet was used as a substrate for the dielectric paste. For this case, the resonator was calibrated while the plastic sheet substrate was inserted into the resonator, instead of using empty resonator. This way, the influence of the plastic sheet on the dielectric measurement was mitigated. It should be noted, however, that no significant difference between the measurement result of a paste sample using an empty resonator calibration or plastic sheet calibration was found. This is understandable, because the plastic sheet has a thickness of approximately 0.1 mm and a much lower dielectric constant and loss tangent as compared to the dielectric paste.

**Figure 2:**
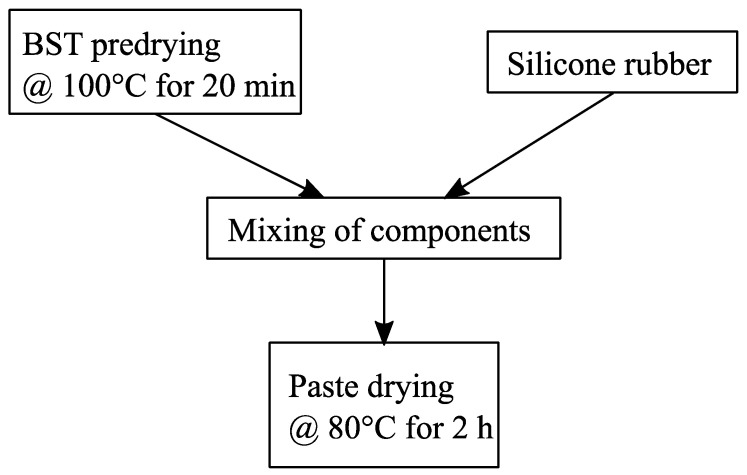
Flowchart of the dielectric paste processing. The BST powder was pre-dried at 100 °C for 20 min. After that, it was thoroughly mixed with the silicone rubber, resulting in a paste with smooth consistency. The resulting paste was then dried again for 2 h at 80 °C or until it was dry.

Next, the preparation of the dielectric paste samples will be described. A flowchart of the procedure is given in Figure 2. Before the two components of the paste, namely, the BST particles and the silicone rubber, were mixed together, the BST particles were predried in an oven for 20 min at 100 °C. This ensured that moisture, which can be absorbed by the BST particles, was evaporated. It is well known in the microwave community that water is a material with a large dielectric loss tangent. Therefore, moisture can affect the dielectric loss of the paste significantly if it is absorbed by the BST powder. After the BST particles were dried, they were mixed with the silicone rubber.

The paste was thoroughly mixed in order to prevent lumps of BST material surrounded by the silicone rubber inside the mixture, and a smooth paste was obtained. The paste was then transferred into the measurement fixture such that the fixture was filled up to the edges, creating a thin layer of dielectric paste. A 3D-printed frame with a thickness of approximately 0.8 mm was used as a boundary to contain the dielectric paste on the plastic sheet. This way, the thickness of the dielectric paste layer was approximately the same as the frame thickness, which is important for the calculation of the dielectric constant and loss tangent of the paste. Next, the paste was dried in an oven at 80 °C for 2 h or until it solidified. After solidification, the paste was ready to be measured in the split resonator setup.

**Figure 3:**
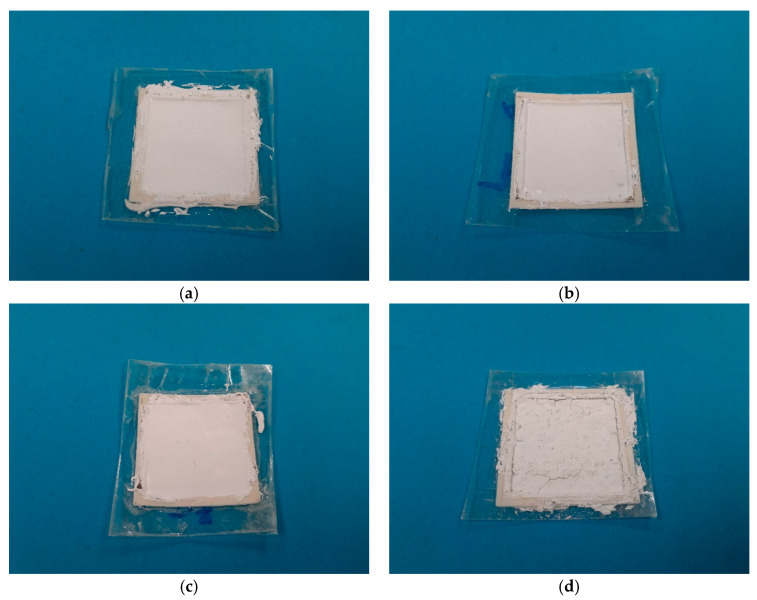
Photographs of four of the fabricated measurement samples of the dielectric paste: (**a**) 40% weight percent BST, (**b**) 60% weight percent BST, (**c**) 70% weight percent BST, and (**d**) 80% weight percent BST. With increasing BST content, the dried dielectric paste became more brittle.

Photographs of dielectric paste samples [27] and their fixtures and substrates for different weight percentages of the BST powder are shown in Figure 3. For relatively low contents of the BST (40%, 60%, and 70%), the paste is smooth, as shown in Figure 3a–c. When the BST content was further increased, the paste started to become increasingly viscous. Eventually, the BST content was so high that the dielectric paste started to have cracks after drying, resulting in poor mechanical properties. This is the case for a BST content of more than 80% and is shown in Figure 3d. A rough surface and cracks can be observed all along the surface of the sample. Due to the increased viscosity in its liquid state, the paste with more than 80% BST weight content was also very difficult to handle, because it turned very sticky, and processing it into a smooth surface was nearly impossible. It was also found that the paste was very brittle after drying for such high BST contents and, thus, may not be suitable for practical applications. On the other hand, for lower weight percentages of the BST powder, the mechanical properties were mainly dominated by the silicone rubber, and thus, smooth surfaces were achieved, as seen from Figure 3a–c.

**Figure 4:**
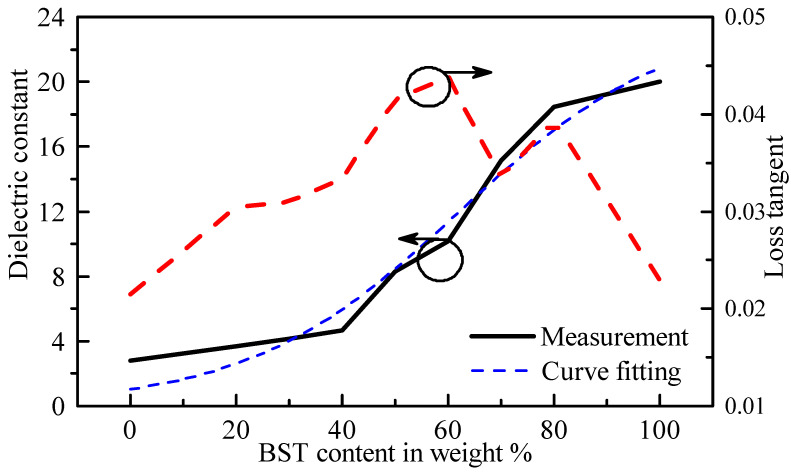
Dielectric constant and dielectric loss tangent of the dielectric paste. The BST content is given in weight percent. A sigmoid curve fit is shown in the graph as well for the dielectric constant of the paste.

Figure 4 shows the result of the measurement of samples of the dielectric paste with different weight content of the BST particles, given in percentage. With reference to the figure, the dielectric constant increases with increasing BST content, which is expected. The lowest and highest BST content, i.e., 0% and 100%, denote the cases of silicone rubber and BST particles only, respectively. A non-linear relationship between the BST weight content and the dielectric constant of the paste was found generally. For lower values of the BST content, the dielectric constant of the paste changes very slowly. In the range of 40–80% BST content, a more rapid increase of the dielectric constant of the paste with increasing BST content is observed. From 80% to 100%, the increase of the dielectric constant with increasing BST content is slowed down again.

The dielectric loss tangent for different dielectric pastes is shown in Figure 4 as well. As can be seen, the dielectric loss increases with a higher amount of weight percent of the BST to a certain degree. This is surprising, because it was found that both the BST and the silicone had similar loss tangents. Therefore, mixing both materials should result in a similar loss tangent, no matter the weight percentage of each constituent.

However, interestingly, the loss tangent of the mixture was somewhat higher than the loss tangent of both individual materials. It is suspected that this could be due to one or several of the following reasons. As mentioned before, the BST powder has the ability to absorb moisture. Therefore, even though the powder is dried before mixing it with the silicone, it can absorb moisture during the drying process of the dielectric paste. It was found that the drying temperature of the silicone must be limited to below 100 °C or the silicone in the paste started to cast a significant number of bubbles, which resulted in a paste with very poor mechanical properties, as well as high surface roughness. Therefore, the drying temperature could not be increased beyond 100 °C. Another possible reason could be that, after mixing both components, the BST powder may have absorbed the solvent, which kept the silicone in its liquid state. Instead of evaporating, some of the solvent may have stayed trapped inside the silicone. According to our experience, this may have led to an increase of the loss tangent of the paste. It was found that dielectric paste samples that were not totally dried usually exhibited higher loss compared to when they were totally dry. As the surface of the paste dried much faster due to its direct contact with air, it might, therefore, have happened that some amount of the solvent was trapped inside the mixture, finally increasing the loss tangent of the paste.

To allow for easy application of the dielectric paste, a sigmoid curve (or logistic curve) was used in order to represent the relationship between the dielectric constant of the paste and the BST content in weight percent:

(1) $$\varepsilon_{r,paste}=\frac{24.02}{1-\exp(-0.05(x-62.2))}$$

where *x* denotes the amount of BST in weight percent that is mixed with the silicone. The curve is fitted to the measured results of the paste’s dielectric constant, as shown in Figure 4. The fitted curve agrees reasonably well with the measured results and could serve as a starting point for determining the desired dielectric constant of the paste depending on the BST content.

## 3. Antenna Design

**Figure 5:**
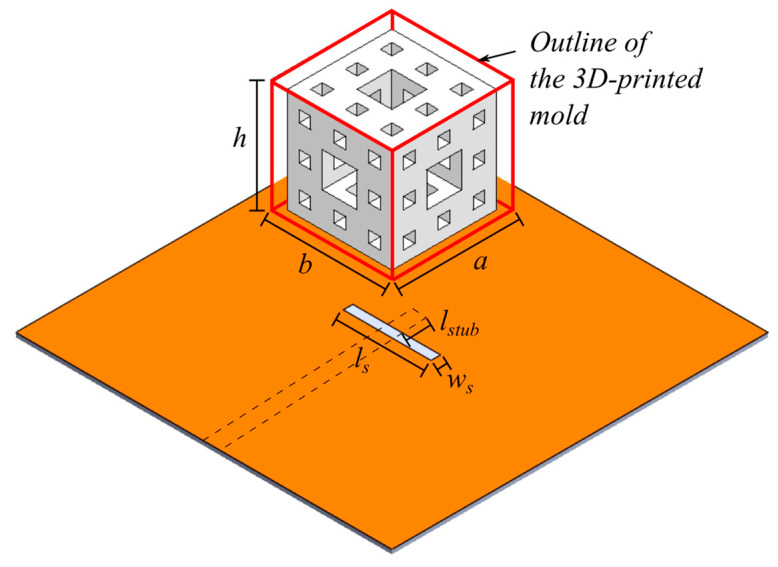
Sketch of the menger sponge fractal DRA in its second iteration The DRA is enclosed by a mold, indicated by the red, solid lines surrounding the DRA. The mold controls the shape of the dielectric paste, which is filled inside and depicted in white color. The DRA has a length, width, and height of *a*, *b*, *h*, respectively. The structure is accommodated by a feeding slot of length and width *l_s_* and *w_s_* and is backed by a microstrip line with width *w_strip_*, which extends the slot by a length *l_stub_*.

In this section, the structure of the antenna, which will serve as an example design for the use of the dielectric paste, will be analyzed. The basic antenna shape is based on the so called menger sponge fractal. An illustration of the second iteration of a menger sponge fractal DRA is shown in Figure 5. A microstripline slot feeding is used to excite the DRA, as indicated by the dashed line in Figure 5. The slot has a length and width of *l_s_* and *w_s_*, respectively. The microstripline has a width of *w_strip_* and extends the slot by a length of *l_stub._* The shape of the menger sponge DRA is defined by an enclosure or mold (depicted by the red solid line in Figure 5), which ensures that the dielectric paste (depicted in white color) in its liquid form stayed in place after being inserted into the mold. The mold was obtained by 3D-printing, and the mold material has a dielectric constant of 3. The inside of the mold was then filled with a dielectric paste of dielectric constant 10.14, corresponding to a dielectric paste with 60% BST content. The DRA has a length, width and height of *a*, *b* and *h*, respectively. The basic shape of the menger sponge fractal consists of a cube with openings inside the dielectric paste in each of the faces, penetrating from one face to the adjacent face on the opposite side of the cube. The openings are realized in practice by using the mold to shape the dielectric paste. The mold itself corresponds to the negative of the shape of the dielectric paste or the menger sponge fractal shape. With an increasing number of iterations of the menger sponge fractal structure, the amount of removed dielectric paste material sections is increased.

**Figure 6:**
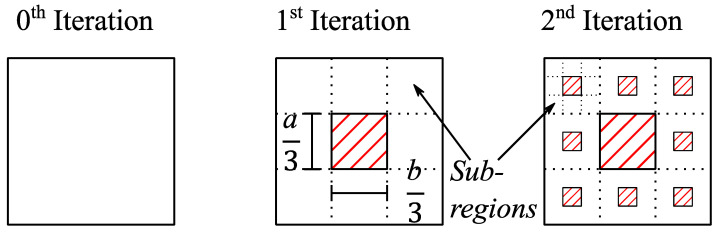
Construction principle of the menger sponge fractal. The zeroth iteration corresponds to the original surface without any modifications. In the first iteration, a slab of material is removed in the center of the face. The cross section of this slab corresponds to one third of side lengths of the rectangle. In the second iteration, the surface of the rectangle is subdivided into nine smaller surfaces with side lengths *a*/3 and *b*/3. For each of those smaller surfaces, the process of the first iteration is repeated, such that a slab of material with cross section *a*/9 and *b*/9 is removed.

The construction principle of the menger sponge fractal for different iterations is illustrated in Figure 6 using a single face of the 3-dimensional structure as an example. For the 0^th^ iteration, the menger sponge fractal corresponds to a cube with no parts of the structure removed.

In the first iteration, however, a rectangular slab of material is removed from the center of each of the faces penetrating through the whole depth of the cube. The removed section has a sidelength of *a*/3 or *b*/3 (or *h*/3), respectively, depending on which side of the cube is considered. Note that other fractions of the side length of the removed sections could be used as well. The removed section penetrates through the whole structure, as is illustrated in Figure 5, effectively removing a slab of material. After the first iteration, each face of the cube can be subdivided into nine new regions, as shown in Figure 6, each of the new regions having the same width and length (for example a/3 and b/3). In the second iteration, each of the new regions has a slab of material removed in their center with a width and length of 1/9 of the original width and length of the large surface or 1/3 of the value of the side lengths of the newly introduced regions. This process can be repeated to reach the next iteration.

**Figure 7:**
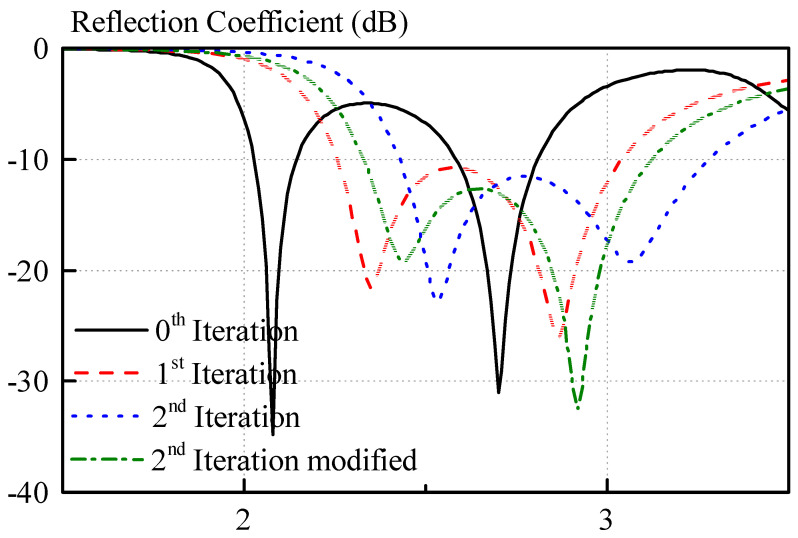
Variation of the reflection coefficient of the menger sponge fractal for different iterations with *a* = *b* = *h* = 25 mm and a dielectric constant of 10.14. The feeding circuit for each iteration is optimized such that maximum bandwidth can be achieved. The feeding parameters for each iteration are: *l_slot,0_* = 20 mm, *l_stub,0_* = 6 mm, *l_slot,1_* = 21 mm, *l_stub,1_* = 6.5 mm, *l_slot,2_* = 21 mm, *l_stub,2_* = 5.5 mm, and *l_slot,2mod_* = 21 mm, *l_stub,2mod_* = 6.5 mm.

A plot of the reflection coefficient of a microstripline slot fed menger sponge fractal DRA, as pictured in Figure 5, for different iterations of the menger sponge fractal is shown in Figure 7. For each of the iterations, the feeding circuit was optimized to maximize the impedance bandwidth performance, whereas the geometrical dimensions of the structure remained unchanged. The feeding parameters for each case are given in the caption of Figure 7. In particular, for each iteration of the menger sponge fractal DRA, the feeding parameters *l_slot_* and *l_stub_* were optimized to achieve the maximum impedance bandwidth. For the 0^th^ iteration, two resonant modes are found spaced far away from each other on the frequency spectrum, and the individual resonance bandwidths are not broad enough to merge the modes to form a wide impedance bandwidth. In particular, the resonances are spaced apart by 0.62 GHz, and the reflection coefficient is larger than −10 dB between the two resonances.

After the first iteration, the two resonant modes are spaced much more closely (approximately 0.52 GHz) and give rise to a much wider impedance bandwidth. The bandwidths for the 0^th^ iteration are 5.3% (2.03 GHz to 2.14 GHz) and 8.5% (2.58 GHz to 2.81 GHz). After the first iteration, the bandwidth was improved to 29.4% (2.26 GHz to 3.04 GHz).

Notably, the individual resonances appear to have a broadened bandwidth, which can be seen from the fact that the reflection coefficient of the individual resonances of the 0^th^ iteration are much narrower on the frequency spectrum. The reflection coefficients of the two resonances of the 1^st^ iteration are not as sharp but vary more slowly with respect to a change in frequency. This can be explained with the quality factor of the DRA. In the structure, at the positions where holes or removed slabs were introduced, the dielectric paste with a dielectric constant of 10.14 was replaced with a material low dielectric constant, in this case a 3D-printed material with a dielectric constant of 3. Thus, the average dielectric constant of the DRA is decreased, leading to a reduction of the quality factor of the DRA, finally, broadening the bandwidth of the individual resonances. After the first iteration, the antenna can only be marginally matched across its passband. In order to achieve better matching, the iteration number can be increased. For the second iteration of the structure, the distance of the resonances on the frequency spectrum is further decreased, and the quality factor is reduced again, as elaborated previously. The modes are spaced more closely, and a better impedance match was achieved throughout the passband of the antenna compared to the first iteration.

**Figure 8:**
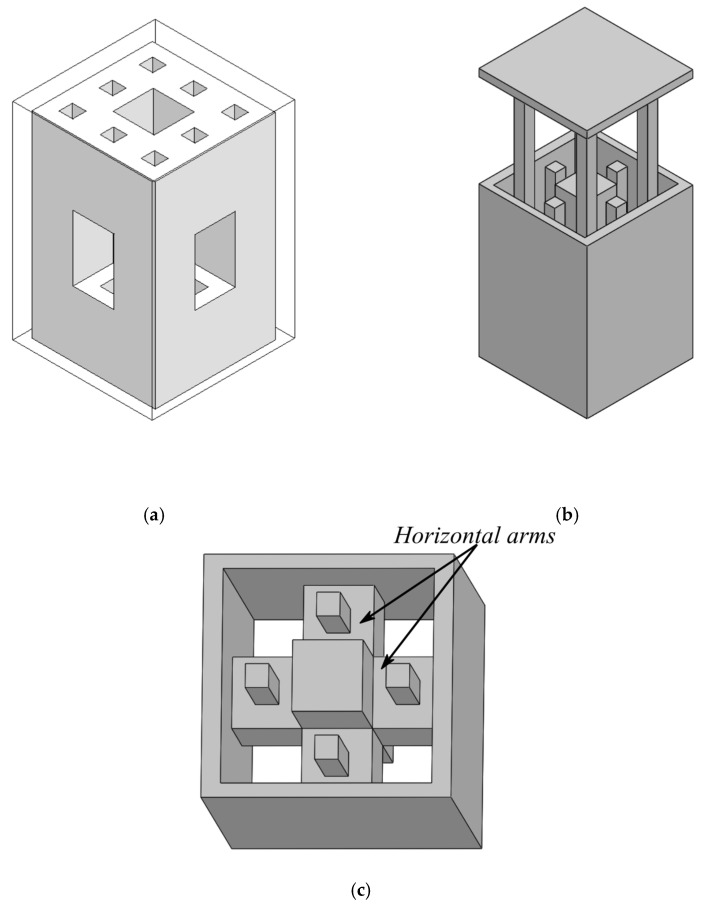
(**a**) Modified menger sponge fractal DRA structure. The dielectric paste is highlighted in white, whereas the outer part of the plastic mold is indicated by a solid line. As compared to the original menger sponge (see Figure 5), the horizontal bars of the second iteration have been removed in order to allow for an easier fabrication. (**b**) Exploded view of the 3D-printed mold structure, which is used for manufacturing process. The mold is split into two parts allowing for an easier manufacturing. The mold is then subsequently filled with the dielectric paste material. (**c**) Perspective view of the inside of the 3D-printed mold showing the horizontal arms that give rise to the “holes” in the dielectric paste in (**a**).

Even though, in this work, the dielectric paste approach is used to construct the DRA, the second iteration of the menger sponge fractal is still too complicated to be realized. Therefore, the structure will be simplified in order to allow for an easier fabrication of a 3D-printed mold. A modified version of the menger sponge fractal structure is shown in Figure 8a. As compared to the second iteration, as shown in Figure 5, the horizontal slabs of 3D-printed material of the second iteration of the menger sponge fractal have been removed in order to ease the fabrication of this structure, whereas all the vertical slabs are retained. Using our in-house 3D-printer, horizontal bars cannot be printed without any supporting material, which would have to be removed after the 3D printing process. Therefore, removing all of the horizontal slabs of the second iteration of the menger sponge fractal DRA guarantees a much easier fabrication of the 3D-printed mold. The mold, which is used to contain the dielectric paste, is shown in Figure 8b. It consists of two separate parts, which are required due to the limitation of the 3D-printing process and in order to reduce the amount of supporting structures. Figure 8c depicts the inside of the 3D-printed mold. The horizontal arms can be seen, which give rise to the “holes” in the dielectric paste structure, as shown in Figure 8a. The reflection coefficient of the modified second iteration excited by a simple slot (compare Figure 5) is also shown in Figure 7. Notably, due to the removal of the horizontal slabs of the menger sponge fractal, the resonance frequencies of both resonant modes are shifted downward, but similar bandwidth and matching compared to the second iteration are achieved. The impedance bandwidth of the linearly polarized, modified menger sponge fractal DRA can cover frequencies ranging from 2.33 GHz to 3.13 GHz, including ISM, WiFi, as well as the WiMAX frequency bands.

**Figure 9:**
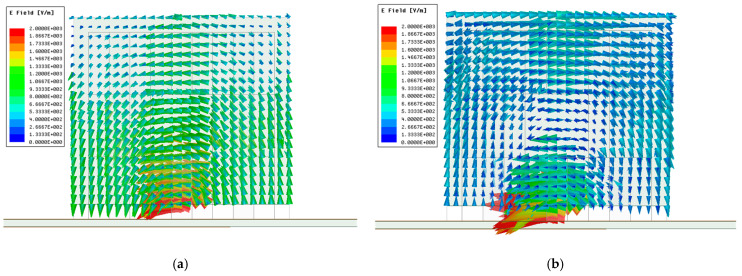
Electric field distributions of modified menger sponge fractal DRA. (**a**) 2.45 GHz; (**b**) 2.9 GHz.

In order to analyze the operating mechanism of the circularly polarized antenna, the resonant modes inside the linearly polarized antenna, excited by a simple slot as shown in Figure 5, are characterized first. The electric field distribution of the modified menger sponge fed by a simple slot are shown in Figure 9a,b at their resonance frequencies at 2.45 GHz and 2.9 GHz (compare Figure 7), respectively. The field distributions at 2.45 GHz indicate that the DRA operates in its fundamental TE*_δ_*_11_ mode. At 2.9 GHz, the field distributions resemble those of the TE*_δ_*_13_ mode.

**Figure 10:**
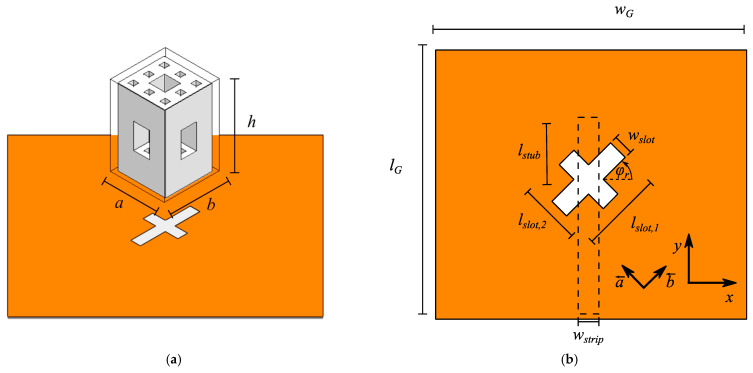
(**a**) Perspective view of the menger sponge fractal from Figure 8, showing its dimensions, along with the feeding mechanism. (**b**) Top view of the feeding PCB with a cross slot with lengths *l_slot,1_*, *l_slot,2_*, and width *w_slot_*. The slot is accommodated by a microstrip feed line with a width *w_strip_* and extends the center of the cross slot by a length *l_stub_*. The ground plane has a length and width of *l_G_* and *w_G_*, respectively. The feeding substrate has a thickness of *t_sub_*. The feeding slot is rotated by an angle of *φ_r_*.

The feeding mechanism of the right hand circularly polarized antenna design, along with the DRA structure, are shown in Figure 10a,b. A cross-slot feeding is used in order to generate the orthogonal modes required for obtaining a circularly polarized antenna. The cross-slot is rotated by an angle *φ_r_* = 45°, and the PCB substrate has a thickness of *t_sub_* = 1 mm.

Using the cross-slot arrangement, orthogonal DRA modes can be excited. In particular, the slot with length *l_slot,1_* excites TE^b^ modes, whereas the slot with length *l_slot,2_* excites TE^a^ modes, where the superscripts *a* and *b* denote the directions along the dimensions *a* and *b* of the DRA, as indicated by the vectors *a* and *b* in Figure 10b. Because the slots have different lengths, the resonance frequencies of the orthogonal modes are slightly different. This translates to the modes resonating with different phases with respect to each other. For the right choice of the slot lengths, the phase difference is approximately 90°, resulting in circular polarization. In this design, each slot excites two DRA modes, namely, TE^a/b^ *_δ_*_11_ and TE^a/b^ *_δ_*_13_, thus, wide axial ratio bandwidth is achieved.

Results for the circularly polarized antenna will be discussed in the measurements section.

## 4. Measurements

**Figure 11:**
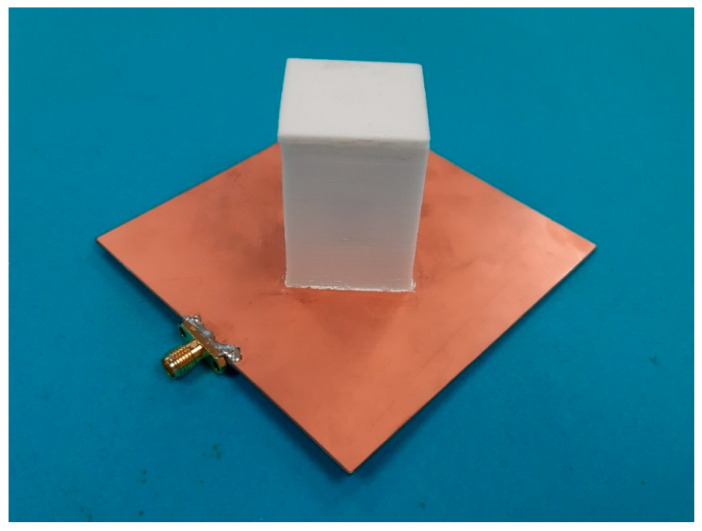
Photograph of the fabricated antenna prototype with the following dimensions: *a* = 25.6 mm, *b* = 25 mm, *h* = 41 mm, *w_slot_* = 5.2 mm, *l_slot,1_* = 32.6 mm, *l_slot,2_* = 18.7 mm, *l_stub_* = 8.5 mm, *l_G_* = *w_G_* = 90 mm, and *w_strip_* = 2.7 mm. A dielectric constant of 10.14 or 60% BST content was used to construct the antenna.

In this section, the measured and simulated results of the antenna prototype will be presented. A photograph of the antenna prototype is displayed in Figure 11, with its final dimensions given in the caption of the figure. The structure of the DRA is the same as the one depicted in Figure 8a. The DRA was attached to the cross-slot feeding using the dielectric paste as a glue in order to prevent air gaps, which could otherwise affect the antenna performance. The feeding PCB has a dielectric constant of 2.65 and a thickness of 1 mm. It should be pointed out that the antenna was constructed in several iterations of a filling and drying process. Because of a much increased volume and thickness compared to the dielectric paste samples, as described in the first section of this publication, longer drying time was necessary. However, the same temperature for the dielectric paste samples was used for drying the BST powder and the dielectric paste after filling the 3D-printed mold. The antenna was made with a dielectric paste with dielectric constant 10.14 or 60% BST content, and the 3D-printed mold had a dielectric constant of 3.

**Figure 12:**
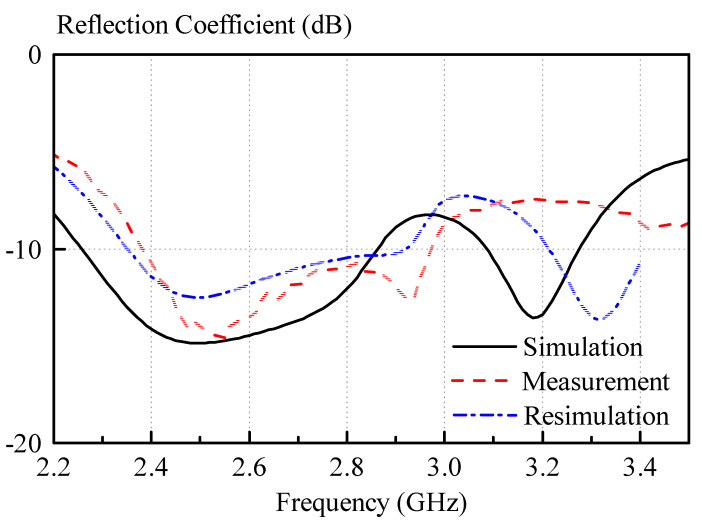
Measured and simulated reflection coefficients. The measured and simulated impedance bandwidth are 21.6% (2.39–2.97 GHz) and 23.4% (2.26–2.86 GHz). For the resimulation, the following values have been used: *a* = 24 mm, *b* = 24 mm, and a dielectric constant of the dielectric paste of *ε_r,paste_* = 9.5.

A comparison of the measured and simulated reflection coefficients of the antenna prototype is given in Figure 12. With reference to the figure, reasonable agreement was obtained, and the measured and simulated impedance bandwidth are 21.6% (2.39–2.97 GHz) and 23.4% (2.26–2.86 GHz), respectively.

**Figure 13:**
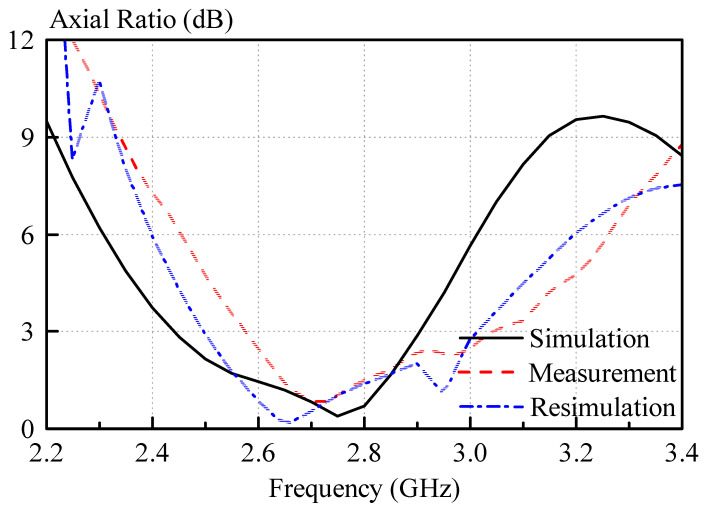
Measured and simulated axial ratios of the right hand circularly polarized antenna. Measured and simulated axial ratio bandwidths of 16.7% and 17.6% were obtained. For the resimulation the following values have been used: *a* = 24mm, *b* = 24mm and a dielectric constant of the dielectric paste of *ε_r,paste_* = 9.5.

Measured and simulated axial ratios are shown in Figure 13. The axial ratios are smaller than 3 dB from 2.575 GHz to 3.05 GHz and 2.44 GHz to 2.91 GHz in measurement and simulation. Again, reasonable agreement was obtained, with measured and simulated axial ratio bandwidths (axial ratio < 3 dB) of 16.7% and 17.6%.

Discrepancies between the measured and simulated reflection coefficients and axial ratios are most likely caused by measurement inaccuracies of paste dielectric constant using the split-post resonator measurement. Even though a 3D-printed frame was used to control the thickness of the dielectric paste samples, it was very difficult to obtain totally flat samples of the dielectric paste in practice. For the measurement of the samples, an average thickness was used for the calculation of the dielectric constant. This leads to inaccuracies in the dielectric constant calculation, because the thickness is one of the inputs for the measurement software. This causes the dielectric paste measurement to be subject to some tolerance in the dielectric constant and loss tangent measurement. Other factors that contribute to the frequency shift of the antenna are the manufacturing tolerances, for instance, caused by the limited accuracy of the 3D printer, which was used in the experiment. However, overall, the results show the feasibility of the dielectric paste approach. The deviations of the measurements from the simulated results are further analyzed in the section “Discussion”.

**Figure 14:**
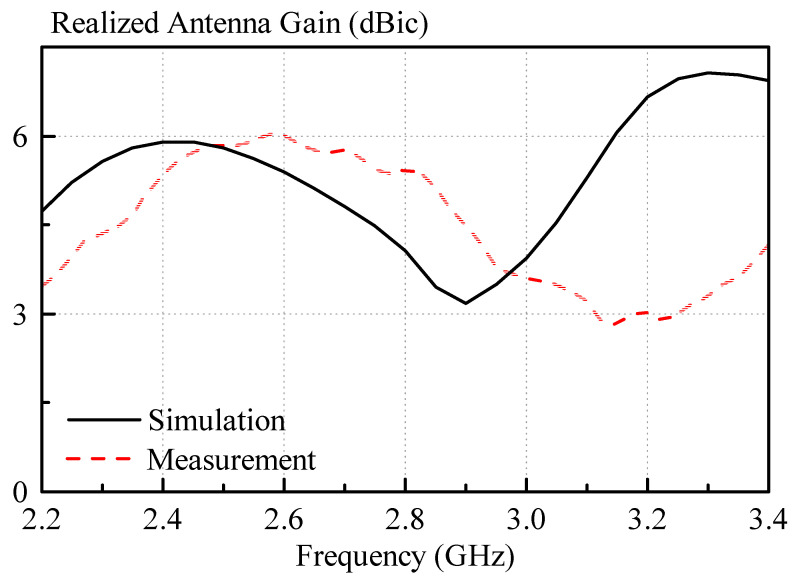
Measured and simulated gain of the right hand circularly polarized antenna.

The measured and simulated broadside gains of the circularly polarized antenna are shown in Figure 14. A maximum measured broadside gain of 6 dBic was found, whereas the simulated counterpart was 5.9 dBic, at 2.6 GHz and 2.4 GHz, respectively.

**Figure 15:**
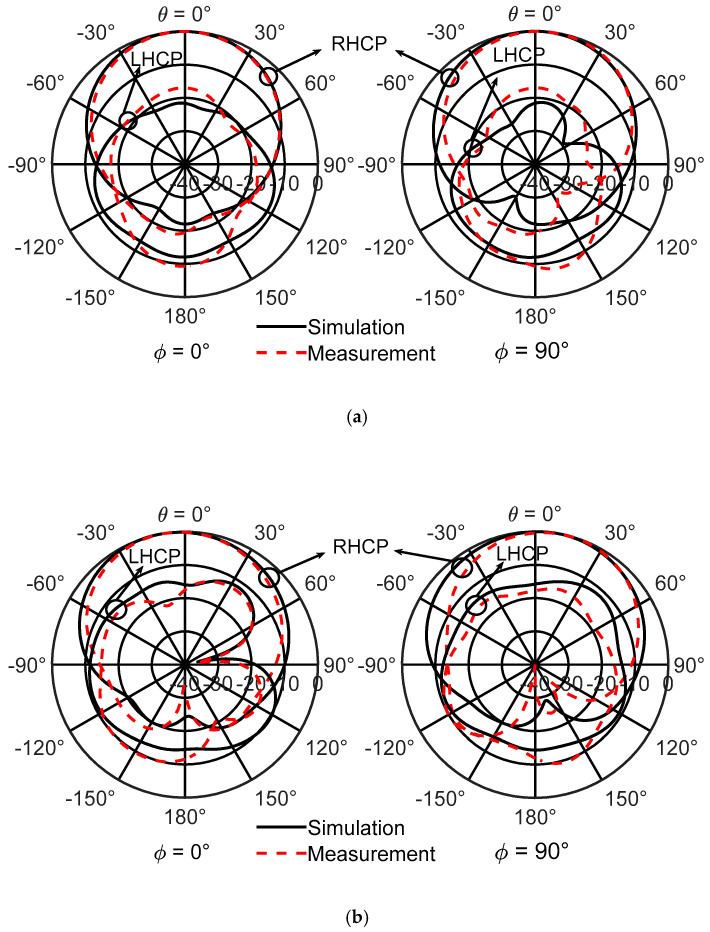
Measured and simulated radiation patterns of the right hand circularly polarized antenna. (**a**) 2.6 GHZ and (**b**) 2.9 GHz.

The measured and simulated radiation patterns are shown in Figure 15a at 2.6 GHz and in Figure 15b at 2.9 GHz for *φ* = 0° and *φ* = 90°, respectively. Good agreement with the simulated results was found, and the backlobe radiation of the antenna is generally much lower than −10 dB. The cross polarization (or left hand circularly polarized radiation) is generally low and close to −10 dB. Stable broadside radiation over the whole antenna passband is observed.

**Figure 16:**
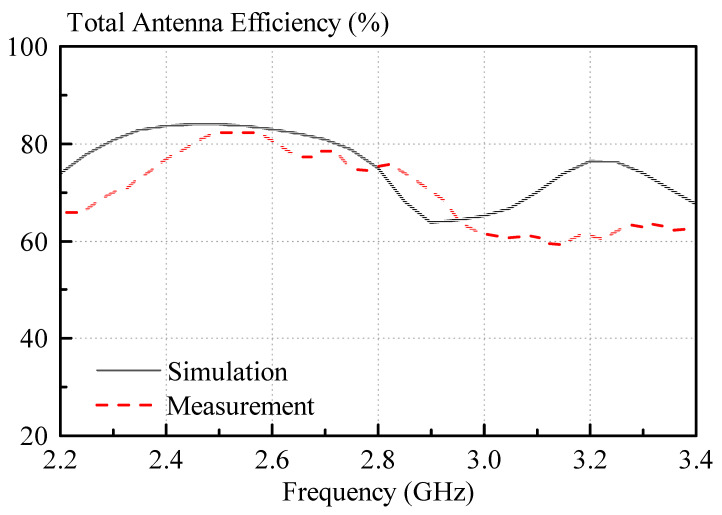
Measured and simulated total antenna efficiencies, including the mismatch of the antennas.

In Figure 16, the measured and simulated antenna efficiencies are displayed. Again, reasonable agreement between simulation and measurement was found. The peak efficiencies for measurement and simulation are 82.3% and 84%, respectively. This is surprisingly large, considering the loss tangent of the dielectric paste. However, it should be noted that the antenna is an effective DRA, consisting of parts with higher and lower dielectric constants, i.e., dielectric paste and the low loss 3D-printed plastic. The 3D-printed plastic lessens the effect of loss tangent of the dielectric paste to some extent.

## 5. Discussion

**Figure 17:**
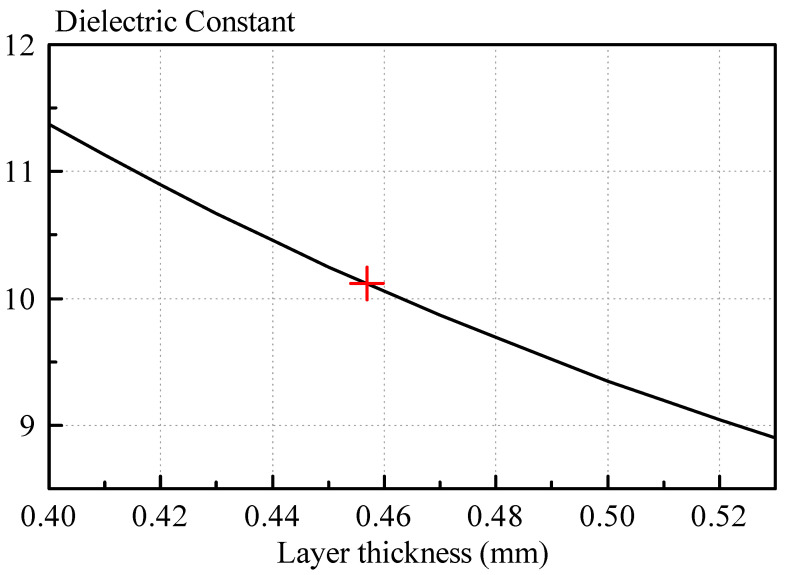
Dielectric constant measurement of the 60% weight percent BST sample with varying input thicknesses for the calculation software. The nominal averaged thickness of the sample is 0.457 mm with a calculated dielectric constant of 10.14.

The dielectric constant of the dielectric paste sample with 60% BST content is shown in Figure 17. The red marker indicates the nominal, calculated dielectric constant of 10.14 for an averaged thickness of the sample of 0.457 mm. Varying the thickness of the sample in the calculation software, the estimated dielectric constant of the sample changes. For a thicker sample thickness, the dielectric constant is reduced, whereas for a thinner thickness, it is increased. For a thickness deviation of ± 0.025 mm, the dielectric constant changes by a value of approximately ± 0.5. Therefore, in practice, a measurement error of the dielectric constant of the order of ± 0.5 should be anticipated.

Furthermore, the dielectric paste samples were not totally flat, causing additional measurement error. The electric field strength in the gap of the SPDR setup in Figure 1 is not homogeneous. In particular, the electric field strength is strongest at the edges of the gap and weakest in the center of the gap. If a sample is warped, part of the sample will be at a location of weaker electric field. Therefore, the resonance frequency of the SPDR will be affected relatively less compared to the case of the flat sample. This will lead to a decrease of the measured dielectric constant.

Lastly, it was found that the 3D-printed mold was suspect to manufacturing tolerance. In particular, the samples were slightly smaller than the values used in the simulation.

The antenna was resimulated, taking into account measurement inaccuracies of the dielectric constant and manufacturing tolerances of the 3D-printed mold, and the results are shown in Figure 12 and Figure 13. Much better agreement with the measured results can be observed for the resimulated antenna for both reflection coefficient, as well as axial ratio.

## 6. Conclusions

In this publication, a new approach for designing dielectric resonator antennas is presented. The method employs a dielectric paste and a 3D-printed mold, which is filled with the dielectric paste material. A dielectric paste can be obtained by mixing BST nano particle sized powder with a silicone rubber. By controlling the BST to rubber ratio, the dielectric constant of the paste can be flexibly controlled with a dielectric constant ranging from 3.67 to 18.45, corresponding to a BST powder content of 20% to 80% in weight percent. The loss tangent of the paste was found to be generally lower than 0.044. Using the dielectric paste, a circularly polarized menger sponge fractal DRA was constructed. The 3D-printing technique was used to obtain a mold structure, which defines the shape of the dielectric paste after it is inserted into the mold and dried. The antenna was found to have an impedance bandwidth of 21.6%, whereas the axial ratio bandwidth was found to be 16.7%, with stable broadside radiation pattern. A peak efficiency of the antenna of 82.3% was obtained by measurement. The measured overlapping bandwidth (reflection coefficient < −10 dB and axial ratio < 3 dB) was found to be from 2.575 GHz to 2.97 GHz or 14%. The measured antenna prototype is suitable for WiMAX applications.

## Data Availability

Not applicable.
